# Association of microRNAs With Embryo Development and Fertilization in Women Undergoing Subfertility Treatments: A Pilot Study

**DOI:** 10.3389/frph.2021.719326

**Published:** 2021-09-23

**Authors:** Alexandra E. Butler, Thomas Keith Cunningham, Vimal Ramachandran, Ilhame Diboun, Anna Halama, Thozhukat Sathyapalan, S. Hani Najafi-Shoushtari, Stephen L. Atkin

**Affiliations:** ^1^Research Department, Royal College of Surgeons Ireland, Adliya, Bahrain; ^2^Academic Diabetes, Endocrinology and Metabolism, Hull York Medical School, University of Hull, Hull, United Kingdom; ^3^The Hull IVF Unit. Women's and Children's Hospital, Hull Royal Infirmary, Hull, United Kingdom; ^4^Division of Research, MicroRNA Core Laboratory, Weill Cornell Medicine-Qatar, Qatar Foundation, Education City, Doha, Qatar; ^5^Hamad Bin Khalifa University (HBKU), Qatar Foundation (QF), Doha, Qatar; ^6^Division of Research, Weill Cornell Medicine-Qatar, Qatar Foundation, Education City, Doha, Qatar; ^7^Department of Cell and Developmental Biology, Weill Cornell Medicine, New York, NY, United States

**Keywords:** microRNA, IVF, fertilization rate, embryo, infertility

## Abstract

**Objective:** Small non-coding RNAs, known as microRNAs (miRNAs), have emerging regulatory functions within the ovary that have been related to fertility. This study was undertaken to determine if circulating miRNAs reflect the changes associated with the parameters of embryo development and fertilization.

**Methods:** In this cross-sectional pilot study. Plasma miRNAs were collected from 48 sequentially presenting women in the follicular phase prior to commencing *in vitro* fertilization (IVF). Circulating miRNAs were measured using locked nucleic acid (LNA)-based quantitative PCR (qPCR), while an updated miRNA data set was used to determine their level of expression.

**Results:** Body mass index and weight were associated with the miRNAs let7b-3p and miR-375, respectively (*p* < 0.05), with the same relationship being found between endometrium thickness at oocyte retrieval and miR-885-5p and miR-34a-5p (*p* < 0.05). In contrast, miR-1260a was found to be inversely associated with anti-Mullerian hormone (AMH; *p* = 0.007), while miR-365a-3p, miR122-5p, and miR-34a-5p correlated with embryo fertilization rates (*p* < 0.05). However, when omitting cases of male infertility (*n* = 15), miR122-5p remained significant (*p* < 0.05), while miR-365a-3p and miR-34a-5p no longer differed; interestingly, however, miR1260a and mir93.3p became significant (*p* = 0.0087/0.02, respectively). Furthermore, age was negatively associated with miR-335-3p, miR-28-5p, miR-155-5p, miR-501-3p, and miR-497-5p (*p* < 0.05). Live birth rate was negatively associated with miR-335-3p, miR-100-5p, miR-497-5p, let-7d, and miR-574-3p (*p* < 0.05), but these were not significant when age was accounted for.However, with the exclusion of male factor infertility, all those miRNAs were no longer significant, though miR.150.5p emerged as significant (*p* = 0.042). A beta-regression model identified miR-1260a, miR-486-5p, and miR-132-3p (*p* < 0.03, *p* = 0.0003, *p* < 0.00001, respectively) as the most predictive for fertilization rate. Notably, changes in detectable miRNAs were not linked to cleavage rate, top quality embryos (G3D3), and blastocyst or antral follicle count. An ingenuity pathway analysis showed that miRNAs associated with age were also associated with the variables found in reproductive system diseases.

**Conclusion:** Plasma miRNAs prior to the IVF cycle were associated with differing demographic and IVF parameters, including age, and may be predictive biomarkers of fertilization rate.

## Introduction

MicroRNAs are small regulatory transcripts (~22 nucleotides long) that act as the major component of the endogenous small interfering RNA machinery. They bind (1) to target messenger RNA (mRNA) transcripts primarily *via* their first eight bases, which are known as recognition elements or seed regions. This interaction ultimately culminates in mRNA degradation and the translational repression of the target gene, a pivotal mechanism that contributes to the post-transcriptional regulation of gene expression (2–5). Furthermore, microRNA (MiRNA) reduces the expression of a multitude of target mRNAs regulating biological and cellular pathways, clinically impacting insulin resistance, glucose intolerance, type 2 diabetes, obesity, and dyslipidemia (6–11). More recently, increasing evidence of miRNA regulation in fertility has emerged, with some showing their expression in the follicular fluid removed at oocyte retrieval during an *in vitro* fertilization (IVF) cycle (12). As human mural granulosa cells and cumulus cells differentiate from a common progenitor during folliculogenesis, differential miRNA expression has been shown between human mural granulosa cells compared with cumulus cells from pre-ovulatory follicles (13) and in patients with differing ovarian reserve levels, particularly in miR-23 (14). Encapsulated miRNAs in follicular fluid following a single IVF cycle showed miR-92 and miR-130b to be overexpressed in the follicular fluid from oocytes that failed to fertilize (15), while miR-888, miR-214, and miR-454 expression were repressed in impaired quality day 3 embryos (15). Others reported differentially expressed follicular fluid miRNAs in women with fertilized vs. non-fertilized oocytes and in those with top quality embryos (16).

Given the novelty of these findings in oocyte development, it was hypothesized that circulating miRNAs at the beginning of the IVF cycle may be predictive of IVF outcome parameters.

## Materials and Methods

### Study Design

This prospective exploratory pilot cohort study was performed in Hull Hospitals from January 2014 to January 2016. Approval was granted by the Yorkshire and the Humber National Research Ethics Service (NRES) ethical committee, UK. All the women in this study provided written informed consent. Sixty women referred to the IVF clinic for subfertility treatments *via* assisted conception were recruited. Of them, 48 Caucasian women were included in the study. Causes of referral included male factor infertility, anovulation, unexplained infertility, and tubal damage. Participants had no medical conditions or illnesses and their only medication was 400 mcg of folic acid daily. Exclusion criteria included those with a body mass index (BMI) >29, those with a homeostatic model assessment (HOMA) value >2, which indicates insulin resistance, and those who had been previously pregnant. The women who presented with amenorrhoea were investigated for polycystic ovary syndrome according to the diagnostic criteria of the Rotterdam consensus, namely, any two of clinical or biochemical evidence of hyperandrogenism (Ferriman–Gallwey score >8 and free androgen index >4.5, respectively), self-reported oligomenorrhea (≤ menses per year) or amenorrhea (no menses for 3 months or more), and polycystic ovaries on transvaginal ultrasound (≥12 antral follicles in at least one ovary or an ovarian volume of ≥10 cm^3^). All women had prolactin, 17-beta hydroxyprogesterone, and thyroid function tests to exclude other confounding factors. Demographic data is shown in Table 1.

**Table 1:** Patient demographics and mean outcome data for the stimulated ovarian cycles for 48 women undergoing *in vitro* fertilization (IVF).

	**Mean (± S.D.)**
Age (years)	31.9 ± 4.6
Body mass index (kg/m^2^)	25.7 ± 3.7
Anti-Müllerian hormone	32 ± 12
Antral follicle count	27 ± 12
Menarche (years)	13.0 ± 1.6
Endometrium at oocyte retrieval (mm)	10.5 ± 1.9
Follicles aspirated	14 ± 5.11
Eggs Retrieved	9.8 ± 5.1
Fertilization rate	0.7 ± 0.25
Cleavage	5.8 ± 3.7
G3D3	3.8 ± 3.1

*G3D3, top quality embryos on day 3 as per Alpha consensus (17)*.

A standard *in vitro* fertilization antagonist protocol was administered as previously described (18). The patients commenced their recombinant follicle-stimulating hormone (rFSH) stimulation on day 2 of their menstrual cycle using either Merional, Watford, UK (Pharmasure) or Gonal-F, Darmstadt, Germany (Merck Serono). A gonadotropin-releasing hormone (GnRH) antagonist (Cetrotide: Merck Serono) was also used to prevent a premature acute rise in luteinising hormone (LH).

The patients underwent ultrasound scans from day 7 to observe the ovarian response to stimulation and scans were repeated every 48 h. The scans were then used to measure the diameters of the follicles, thus observing response and follicle numbers. Final maturation was triggered when two or more leading follicles were ≥18 mm using a human chorionic gonadotrophin (hCG), Pregnyl, Kenilworth, NJ USA (Merck Sharp and Dohme).

Transcervical embryo transfer was performed, and embryos were classified using standard criteria (19) for cleavage stage (days 2–3 after egg collection) and for blastocyst stage (days 5–6 after egg collection). Top quality embryos were determined on day 3 as per Alpha consensus (17). Embryo transfer was performed on either day 3 or, ideally, at day 5 (blastocyst) to give the best chance for implantation, as this timing is similar when compared to natural cycle embryos moving into the uterus.

The primary endpoint was the association of plasma miRNA measurement and IVF outcome.

### Sample Collection

Just prior to an *in vitro* fertilization cycle, following an overnight fast, blood was drawn in the follicular phase of the menstrual cycle into ethylenediamine tetraacetic acid (EDTA)-containing tubes. Plasma was stored frozen at −80^O^C until batch analyses. Anti-Müllerian hormone measurement was then performed using a Beckman Coulter Access automated immunoassay, with a between-run precision <3% (20).

### miRNA Profiling and Analysis

Ribonucleic acid was prepared and microRNAs measured as previously described (12). Briefly, total RNA was isolated from 200 μl of plasma of each sample using the miRCURY RNA Isolation Kit - Biofluids Exiqon, Qiagen, Germantown MD, USA, acquired by Qiagen following the manufacturer-recommended protocol. Since the precise estimation of RNA concentration from biofluids such as plasma is difficult (21), a reverse transcription was performed with equal volumes of RNA from each sample (4 μl of RNA in 20-μl reverse transcription reaction volume) using an Exiqon Universal cDNA Synthesis Kit II, Qiagen, Germantown MD, USA following the protocol of the manufacturer. Such a method of inputting equal volumes of total RNA into the cDNA synthesis reaction has been described earlier (22). Afterward, RNA integrity and reverse transcription efficiency were assessed using representative samples that were loaded onto an miRNA QC PCR Panel (Exiqon), which included synthetic RNA isolation spike-ins, UniSp2, 4, and 5 (Exiqon) that were added prior to RNA isolation and the cDNA synthesis spike-ins. The Exiqon Serum or Plasma Focus microRNA PCR Panel used for miRNA profiling in this study contained primer sets for 179 circulating miRNAs in addition to assays for synthetic spike-ins and reference miRNAs. Amplification was performed using a QuantStudio 12K Flex Real-Time PCR System, Thermo Fisher, Waltham, MA, USA (ThermoFisher Scientific) followed by the pre-processing of raw data and a statistical analysis using the GenEx qPCR analysis software, version 6 (MultiD). False positive amplifications were eliminated by running a no-template negative control panel and then setting a ΔCt of 1 between the sample and negative control for every miRNA assayed. To identify and leave out haemolysed samples, a ΔCt > 7 between hsa-miR-23a-3p and hsa-miR-451a was set as a cut-off as described (23, 24). Data was then normalized against the global mean of all expressed miRNAs with a Ct less than 35.

### Statistics

No data on specific microRNA on *in vitro* fertilization outcomes were available to undertake a formal power analysis. Therefore, this study was deemed to be a pilot study. Birkett and Day (25) suggest that at least 20 degrees-of-freedom allow the estimation of variance to power a larger study; thus, 24 subjects for each cohort were recruited. Statistics were performed with Statistical Package for the Social Sciences (SPSS) (v22, Chicago, Illinois). Descriptive data were presented as mean ± SD.

A regression model was used to assess the association with each measured microRNAs (incorporated as the y-variable) and demographics in addition to *in vitro* fertilization outcomes (used as x-explanatory variables). Furthermore, the cohort was randomly separated into training and prediction sets of equal sizes. A beta-regression model was then used to regress the fertilization rate (in this case, serving as the y-variable and assumed to follow a beta distribution) on all measured miRNAs with no missing values. The model formula was fertility rate ~ miRNA1 + miRNA2 + miRNA3 +…..+miRNAn + Age + BMI. The model was fit on the training set and iteratively refined to find the smallest set of potentially predictive miRNAs with independent effects. The model was then validated on the prediction set. The regression analysis was conducted in R version 4.0.2.

### Ingenuity Pathway Analysis

The Ingenuity Pathway Analysis (IPA) software (Qiagen, Germantown, Maryland, USA) supports the data analysis and integration of data derived from diverse experimental datasets, including gene expression and miRNA. In this study, the IPA was performed to illustrate the canonical pathways related to the miRNAs highlighted in this study.

## Results

Subject demographics are shown in Table 1 and the microRNAs detected are shown in Supplementary Table 1 (https://doi.org/10.6084/m9.figshare.13525376.v1). The BMI and weight were positively associated with let7b-3p and miR-375, respectively (*p* < 0.05), while miR-1260a was negatively associated with anti-mullerian hormone (AMH; *p* = 0.007) (Table 2). Endometrium thickness at oocyte retrieval was also associated with miR-885-5p and miR-34a-5p (*p* < 0.05). MiR-365a-3p, miR-122-5p, and miR-34a-5p were correlated with embryo fertilization rates (*p* < 0.05). Age was negatively associated with miR-335-3p, miR-28-5p, miR-155-5p, miR-501-3p, and miR-497-5p (*p* < 0.05). Live birth rate was negatively associated with miR-335-3p, miR-100-5p, miR-497-5p, let7d, and miR-574-3p (*p* < 0.05), but these were not significant when age was accounted for.

**Table 2:** Association of miRNA with demographic parameters, anti-Mullerian hormone (AMH), and IVF parameters determined.

**Clinical Parameter**	**miRNA**	**Estimate**	***P*-value**
BMI	hsa.let.7b.3p	0.124	0.050
Weight	hsa.miR.375	0.024	0.039
Age	hsa.miR.335.3p	−0.178	0.020
	hsa.miR.28.5p	−0.143	0.029
	hsa.miR.155.5p	−0.142	0.030
	hsa.miR.501.3p	−0.095	0.044
	hsa.miR.497.5p	−0.059	0.050
AMH	hsa.miR.1260a	−0.017	0.007
	hsa.miR.34a.5p	0.030	0.043
Endometrial thickness at oocyte retrieval	hsa.miR.885.5p	0.042	0.020
	hsa.miR.34a.5p	0.030	0.043
Fertilization rate	hsa.miR.365a.3p	2.746	0.017
	hsa.miR.122.5p	2.430	0.021
	hsa.miR.34a.5p	1.634	0.035
Live birth	hsa.miR.335.3p	−2.542	0.029
	hsa.miR.100.5p	−1.482	0.029
	hsa.miR.497.5p	−0.913	0.045
	hsa.let.7d.3p	−1.248	0.047
	hsa.miR.574.3p	−1.656	0.048

*Common miRNA between age and live birth (yellow highlights), between body mass index (BMI) and live birth (green highlights), and between AMH and endometrial thickness at oocyte retrieval and fertilization rate (blue highlights) are highlighted*.

*A regression model was used to assess the association with each measured miRNA (incorporated as the y-variable) and demographics in addition to IVF outcomes (used as x-explanatory variables). Furthermore, the cohort was randomly separated into training and prediction sets of equal sizes. A beta-regression model was used to regress the fertilization rate (in this case, serving as the y-variable and assumed to follow a beta distribution) on all measured miRNAs with no missing values. The model formula was fertility_rate ~ miRNA1 + miRNA2 + miRNA3 +…..+ miRNAn + Age + BMI. The model was fit on the training set and iteratively refined to find the smallest set of potentially predictive miRNAs with independent effects. The model was then validated on the prediction set. The regression analysis was conducted in R version 4.0.2*.

*BMI, Body Mass Index; AMH, Anti-Mullerian Hormone*.

A beta-regression model regressing the fertility rate on microRNA measurements from the training set identified miR-1260a, miR-486-5p, and miR-132-3p (with an effect size/*p* = −0.41/0.0285,0.87/0.0000023, and −0.77/0.00031, respectively). Since the model in question featured these miRNAs as x-explanatory variables, their calculated effects were independent of each other. It should be noted that the miRNA with the most significant association with the fertility rate from the simple regression (refer to Methods), miR-365a-3p, appeared to lose its effect when assessed together with the rest of the miRNAs in the beta-regression model.

**Figure 1:**
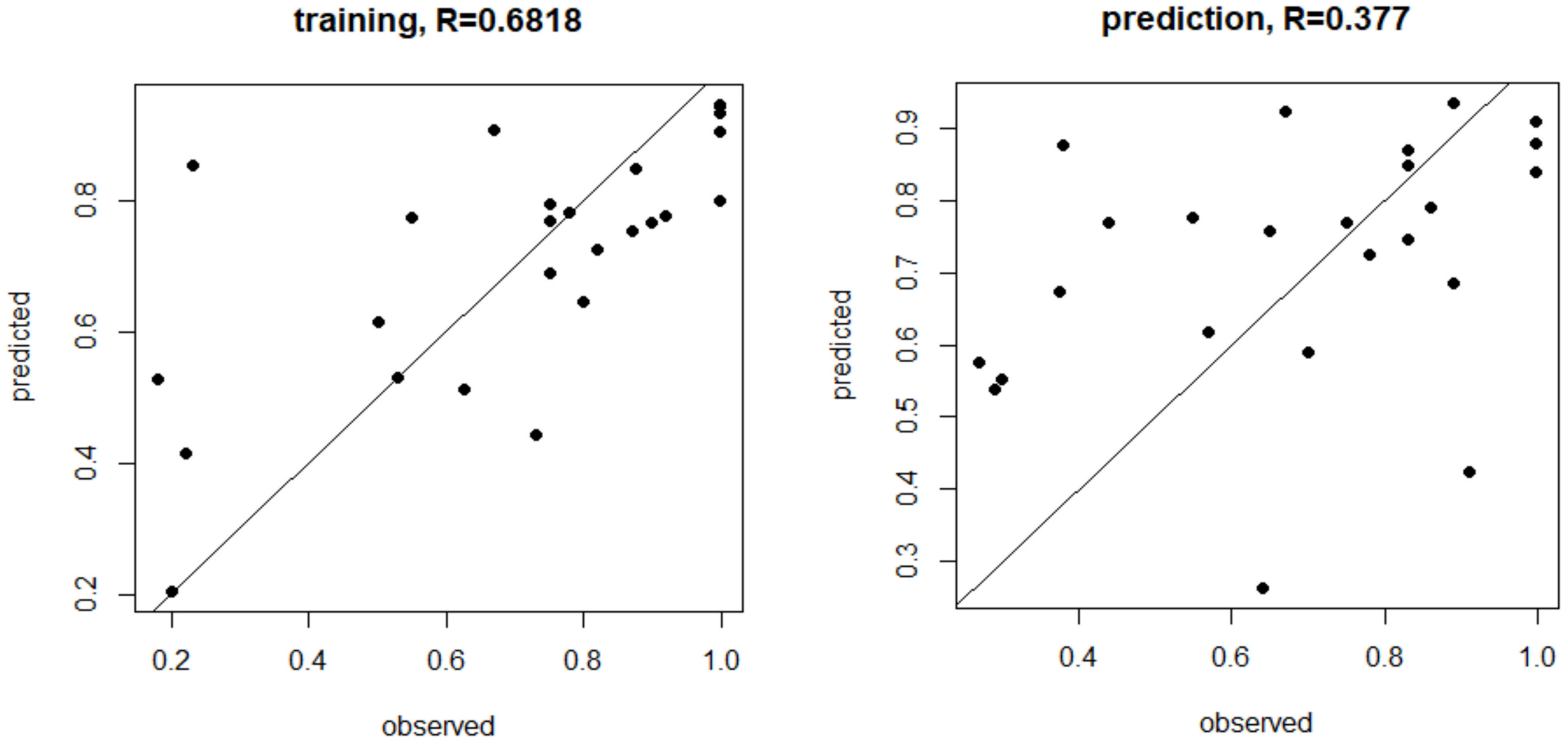
MicroRNA (miRNA) trained and prediction models for live birth rate. The miRNA trained and prediction models for live birth rate show the moderate training effect, but a weaker prediction effect, based on miR-1260a, miR-486.5p, and miR-132.3p (*p* < 0.03, *p* = 0.0003, *p* < 0.00001, respectively, was most predictive of fertilization rate.

Figure 1 shows how the observed fertility rates from the training were correlated with those predicted by the model with the prediction being less robust, though this was expected because the model was trained on the same data.

There was no association of the miRNAs with cleavage rate, top quality embryos (G3D3), and blastocyst or antral follicle count.

Those women in whom the cause of their infertility was the male factor may be considered to be “normal controls” and, therefore, the 15 women with this diagnosis were excluded and reanalysis was undertaken. As noted above, miR-365a-3p, miR122-5p, and miR-34a-5p correlated with embryo fertilization rates (*p* < 0.05). However, after omitting cases of male infertility, miR122-5p remained significant (*p* < 0.05) but miR-365a-3p and miR-34a-5p were no longer so (*p* = 0.08/0.07, respectively). Interestingly, with the omission of the women with male factor infertility, miR1260a and mir93.3p (*p*-values previously = 0.323/0.46) became significant (*p* = 0.0087/0.02, respectively).

As noted above, live birth rate was negatively associated with miR-335-3p, miR-100-5p, miR-497-5p, let-7d, and miR-574-3p (*p* < 0.05). HHHowever, when women with male factor infertility were excluded from the analysis for live birth rate, these miRNAs all lost significance (*p* > 0.05), though miR.150.5p (previously *p* = 0.26) then emerged as significant (*p* = 0.042).

**Figure 2:**
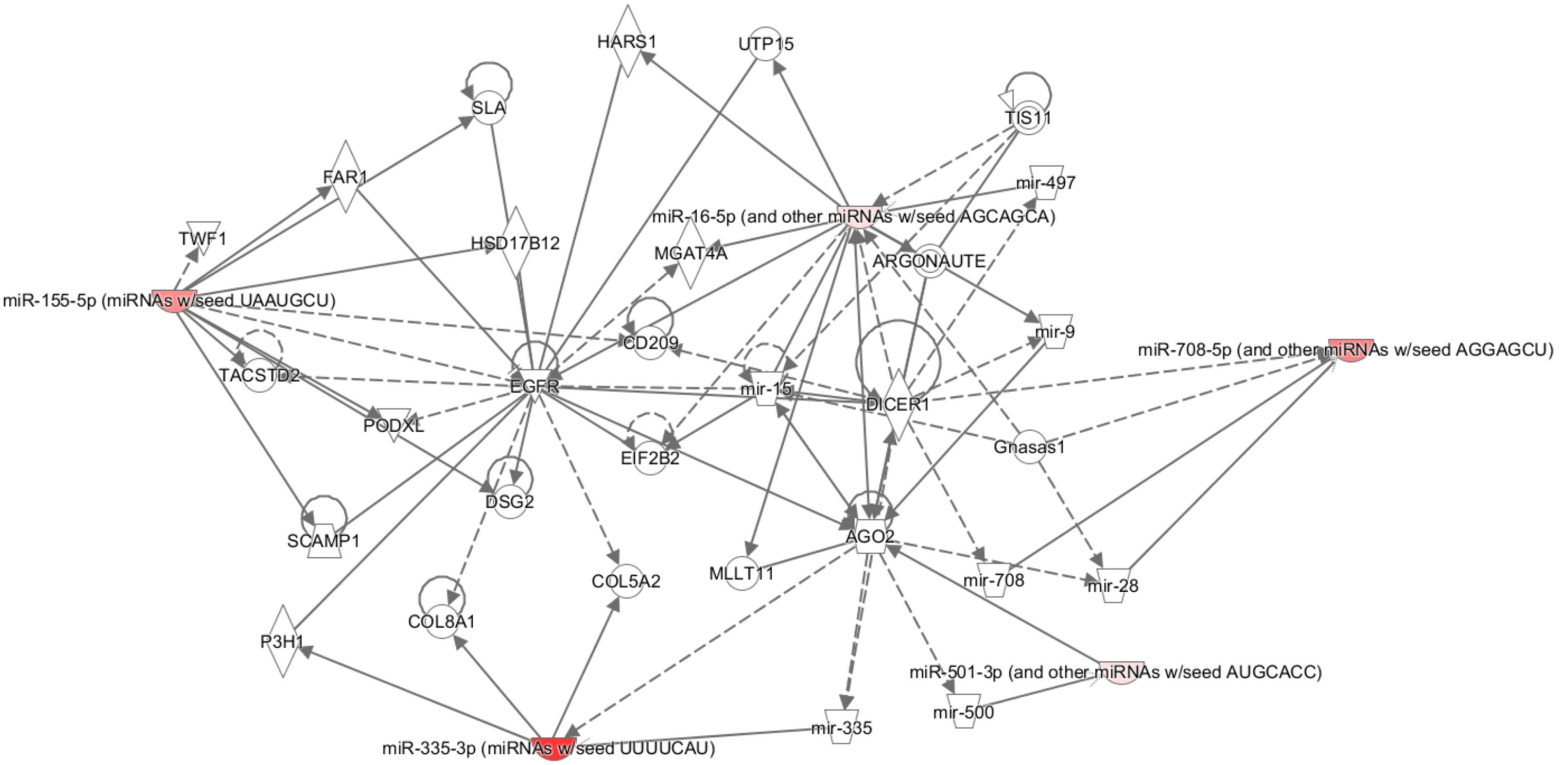
Ingenuity pathway analysis (IPA). Pathway connections for the five miRNAs (miRNAs miR-335.3p, miR-28.5p, miR-155.5p, miR-501.3p, and miR-497.5p) that were significantly associated with age and reproductive abnormalities. The IPA demonstrates the most significantly affected pathways relating to the proteins in question. The miRNA in red showed a significant decrease.

MicroRNAs have been shown to interact in a coordinated manner to create regulatory networks involving other non-coding RNAs (26). Therefore, the IPA was performed for the miRNAs associated with age, fertility rate, follicles aspirated, and live birth. This IPA revealed that four of the five miRNAs associated with age were associated with those found in reproductive system diseases (Figure 2). Furthermore, the IPA analysis showed commonality to the broad group of Organismal Injury and Abnormalities; however, there were no overlapping pathways to identify the common proteins involved (data not shown).

## Discussion

MicroRNAs measured just prior to an *in vitro* fertilization cycle showed that fertilization rate was associated with miR-365a-3p (not previously described), the liver-specific miR-122-5p (not previously described), and miR-34a-5p, whose increased expression was associated with eight-stage embryo formation (27). Omitting cases of male infertility, miR122-5p remained significant but miR-365a-3p and miR-34a-5p were no longer significant (*p* = 0.08/0.07, respectively). However, this small shift in the *p*-value was probably due to a smaller “n” as the study lost power. What was of particular interest was that the new miRNAs only became significant when the male infertility factor women were omitted, specifically miR1260a and mir93.3p, implying that these miRNAs are only significant when the infertility is in the female or that these miRNAs are markers of pre-existing pathologies that have now been exposed.

However, when all the microRNAs detected in the entire panel were used in a beta-regression model of fertilization rate, it showed that miR-1260a, miR-486-5p, and miR-132-3p were most predictive of fertilization rate, though the correlation in the predictive model was not strong. For those miRNAs that were predictive of fertilization rate, namely, miR-486-5p, miR-132, and miR-1260a, many of the pathways highlighted are functionally linked to innate immune response and metabolic disorders, given the fact that most plasma miRNAs either originate from immune and/or liver cells. In case of miR-486-5p, insulin signaling and resistance are linked to hepatic glucose output and increased steatosis. Foxo1 is a putative target of miR-486-5p based on a Targetscan prediction (28, 29), with an interaction being reported in the context of kidney disease. Increased hepatic miR-486 expression may also reduce Foxo1, resulting in reduced insulin resistance that may then affect fertility (30). Furthermore, miR-486-5p was shown to be downregulated in oocytes in polycystic ovarian syndrome (PCOS), which potentially leads to cumulus cell proliferation (31), but increased levels were associated with endometriosis (32). The signaling pathway involving miR-132 is also related to immune cells and inflammation, with some reports showing that miR-132 is protective and reduces inflammation (33, 34). Notably, miR-132-3p is upregulated in follicular fluid and has been linked to top quality embryos, whereas an ingenuity pathway analysis shows that miR-132-3p targets are associated with Reproductive System Disease (16). MiR-132-3p has also been shown to attenuate steroid production *in vitro* (35) and may mediate the GnRH activation of FSH expression (36). In the case of miR-1260a, there are no strong studies functionally linked to fertility, as mir-1260 is associated with proliferative states such as cancer (37), has been identified in human stem cells (38), and is associated with type 2 diabetes (39). In this study, it also showed a negative association with AMH. Overall, this suggests that differing miRNAs, alone or in combination, may have predictive value for fertilization rates that requires both the confirmation and determination of the reproductive functions of these miRNAs.

Five microRNAs were each negatively associated with age, namely, miR-335-5p, miR-28-5p, miR-155-5p, miR-501, and miR-497. An IPA suggested that these are broadly related in reproductive system diseases, but their reproductive functions need clarifying. Furthermore, changes in miRNAs are in accordance with reports that show miRNAs to be altered with aging (40). In particular, miR-335-5p has been shown to negatively regulate granulosa cell proliferation (41) and, in animal models, is important for meiosis (42). MiR-28-5p is significantly lower in euploid blastocysts from women in their forties compared with those from young oocyte donors (43), which is in accordance with its relation to age in this study, and is associated with ovarian and other cancer progressions (44). MiR-155-5p may have a role in aging and age-related diseases and has been shown to be upregulated in the granulosa cells of women with reduced ovarian reserves (45). It also appears to be important in the fibrotic process (46). MiR-501 has not been related to any specific fertility or aging functions. However, it was shown to be increased following the exposure of umbilical cells to estradiol, suggesting how estrogen may modulate endothelial function (47). MiR-497 has been shown to be downregulated in cellular senescence in animal models (48), again relating this miRNA to an association with age as shown here.

Live birth was associated negatively with five microRNAs, namely, miR-335-3p (also seen with age), miR-100-5p, miR-497-5p (also seen with age), let-7d-3p, and miR-574-3p. It is likely that these are surrogate markers for other underlying processes and, when accounted for by age, they were no longer found to be related. In a study looking at implantation markers, no differences in miRNAs were found in euploid blastocysts that did or did not implant (49). With the exclusion of male factor infertility, all these miRNAs lost significance, suggesting that these miRNAs are not predictive of live birth rates, though miR.150.5p then emerged as weakly correlated with live birth. This is interesting in light of the association of miR.150.5p with pregnancy-induced hypertension (50), its involvement in placental function (51), and its association with endometriosis (52).

Let-7b-3p was positively related to body mass index in these normal weight subjects, but no correlation was shown in obese subjects (12). Despite this, it has been shown in animal models that the let-7 axis is associated with weight and female growth (53). Let-7b-3p also has an association with several forms of cancer including melanoma, lung cancer, and ovarian serous carcinoma (genecards.org). Furthermore, weight was positively associated with miR-375, while it has been shown that both let-7 and miR-375 regulate insulin exocytosis and are associated with type 2 diabetes (54). Infertility is also associated with abnormal lipid levels, obesity, and metabolic disorders (55), with miRNAs having been reported to be linked between infertility and metabolism (56, 57).

MiR-34a-5p has been reported to have adverse effects on folliculogenesis in animal models (58) and positively associations with AMH, endometrial thickness, and fertilization rate.

Endometrial thickness has been associated with microRNAs, particularly miR-449a, which may affect endometrial receptivity (59), but there have been no reports associating miR-885 or miR-34a with the endometrium. On the other hand, miR-885-5p has been associated with pancreatic neoplasia.

Others have demonstrated microRNAs as potential biomarkers associated with fertility, with miRNA changes noted in the endometrium (60) in those with recurrent implantation failures (61) and in the follicular fluid of subjects undergoing IVF (15, 62), though this is the first study to determine predictive values of miRNA in IVF. These observations require further clarification and validation, but raise the intriguing possibilities for the measurement of miRNA prior to a fertilization cycle to predict those that may respond best and identify those that may need additional support. For example, the measurement of miR-1260a, 486-5p, and 132-3p giving a positive prediction of increased fertility rate may lower the dosage regimen of the medication in the IVF cycle. The measurement of miR-885 and 34a may also give predictions on endometrial health or receptivity. The measurement of miR-335-3p, miR-100-5p, miR-497-5p, let-7d-3p, and miR-574-3p was negatively associated with live birth but accounted for by age, which may indicate that, above a particular age cut-off, a high-risk pregnancy may result.

There are several limitations of the study that include the derivation of the circulating miRNA. As noted above in the discussion on the differing miRNA, they result from many different tissues and cells, but are not limited to the reproductive system. Therefore, functional studies on the miRNA highlighted need to be undertaken to understand their potential predictive utility. Furthermore, this study was undertaken on a small number of subjects; thus, it was not powered to account for differing causes of infertility, which would require a much greater sample size given the number of IVF parameters and miRNAs measured. Moreover, the women in this study were all Caucasian women, so ethnic differences could not be taken into account.

In conclusion, plasma microRNAs prior to the *in vitro* fertilization cycle were associated with differing demographic and IVF parameters, including age, and may have predictive utility for the fertilization rate.

## Data Availability Statement

The raw data supporting the conclusions of this article will be made available by the authors, without undue reservation.

## Ethics Statement

The studies involving human participants were reviewed and approved by The Yorkshire and The Humber NRES ethical committee, UK. The patients/participants provided their written informed consent to participate in this study.

## Author's Note

SA is the guarantor of this work and, as such, had full access to all the data in the study, thus taking responsibility for the integrity of the data and the accuracy of the data analysis.

## Author Contributions

AB contributed to the data analysis and wrote the manuscript. VR and SN-S performed the miRNA measurements. VR and TC contributed to the data analysis. TS contributed to the study design and supervised the sample collection. ID undertook the statistical analysis. AH performed the ingenuity pathway analysis. SA designed the studies, supervised the work, contributed to data analysis, and was involved in preparation of the manuscript. All authors read and approved the final manuscript.

## Conflict of Interest

The authors declare that the research was conducted in the absence of any commercial or financial relationships that could be construed as a potential conflict of interest.

## Publisher's Note

All claims expressed in this article are solely those of the authors and do not necessarily represent those of their affiliated organizations, or those of the publisher, the editors and the reviewers. Any product that may be evaluated in this article, or claim that may be made by its manufacturer, is not guaranteed or endorsed by the publisher.
