# Corrigendum: Association of microRNAs With Embryo Development and Fertilization in Women Undergoing Subfertility Treatments: A Pilot Study

**DOI:** 10.3389/frph.2022.896196

**Published:** 2022-03-29

**Authors:** Alexandra E. Butler, Thomas Keith Cunningham, Vimal Ramachandran, Ilhame Diboun, Anna Halama, Thozhukat Sathyapalan, S. Hani Najafi-Shoushtari, Stephen L. Atkin

**Affiliations:** ^1^Research Department, Royal College of Surgeons Ireland, Adliya, Bahrain; ^2^Academic Diabetes, Endocrinology and Metabolism, Hull York Medical School, University of Hull, Hull, United Kingdom; ^3^The Hull IVF Unit. Women's and Children's Hospital, Hull Royal Infirmary, Hull, United Kingdom; ^4^Division of Research, MicroRNA Core Laboratory, Weill Cornell Medicine-Qatar, Qatar Foundation, Education City, Doha, Qatar; ^5^Hamad Bin Khalifa University (HBKU), Qatar Foundation (QF), Doha, Qatar; ^6^Division of Research, Weill Cornell Medicine-Qatar, Qatar Foundation, Education City, Doha, Qatar; ^7^Department of Cell and Developmental Biology, Weill Cornell Medicine, New York, NY, United States

**Keywords:** microRNA, IVF, fertilization rate, embryo, infertility

In the published article, there was an error in affiliation 1. Instead of “Research Department, Royal College of Surgeons Ireland, Dublin, Ireland,” it should be “Research Department, Royal College of Surgeons Ireland, Adliya, Bahrain.”

The authors apologize for this error and state that this does not change the scientific conclusions of the article in any way. The original article has been updated.

## Publisher's Note

All claims expressed in this article are solely those of the authors and do not necessarily represent those of their affiliated organizations, or those of the publisher, the editors and the reviewers. Any product that may be evaluated in this article, or claim that may be made by its manufacturer, is not guaranteed or endorsed by the publisher.

