# Supplementary material for: Association of microRNAs With Embryo Development and Fertilization in Women Undergoing Subfertility Treatments: A Pilot Study
Source: Front Reprod Health. 2021 Sep 23;3:719326. doi: 10.3389/frph.2021.719326 (PMC9580729; doi:10.3389/frph.2021.719326)
Supplement: Supplementary file 2 [file Data_Sheet_2.PDF]

INGENUITY
PATHWAY ANALYSIS

Analysis Name: miR-486-5p targets - 2021-08-01 05:19 PM
Analysis Creation Date: 2021-08-01
Build version: exported
Content version: 65367011 (Release Date: 2021-06-04)

Experiment Metadata

Name	Value

Analysis Settings

Reference set: Ingenuity Knowledge Base (Genes Only)
Relationship to include: Direct and Indirect
Includes Endogenous Chemicals
Optional Analyses: My Pathways My List

Filter Summary:
Consider only molecules and/or relationships where
(species = Mouse OR Uncategorized OR Rat OR Human) AND
(confidence = Experimentally Observed) AND
(tissues/cell lines = Trigeminal Ganglion OR Lymph node OR Cornea OR Placenta OR SK-MEL-2 OR U251 OR White Matter OR RPMI-8266 OR SN12C OR OVCAR-8 OR Skeletal Muscle OR OVCAR-4 OR Other Dendritic cells OR Granule cells OR Murine NKT cells OR Medulla Oblongata OR Th2 cells OR Vd2 Gamma-delta T cells OR Nucleus Accumbens OR Granulocytes not otherwise specified OR Other Melanoma

(c) 2000-2021 QIAGEN. All rights reserved.

Cell Lines OR Neutrophils OR Other Cell Line OR CD34+ cells OR Cytotoxic T cells OR BT-549 OR Colon Cancer Cell Lines not otherwise specified OR LOX IMVI OR Striatum OR MDA-N OR Cos-7 cells OR Beta islet cells OR Lens OR Melanocytes OR Other Cells OR SF-539 OR Fibroblasts OR Other Stem cells OR TK-10 OR Bone marrow-derived macrophages OR Granulosa cells OR Forestomach OR MOLT-4 OR MG-63 OR CD56dim NK cells OR EKVX OR Central memory cytotoxic T cells OR Other Memory T lymphocytes OR Other Mononuclear leukocytes OR Cells not otherwise specified OR Lung Cancer Cell Lines not otherwise specified OR T lymphocytes not otherwise specified OR RBL-2H3 OR Caudate Nucleus OR Plasma cells OR K-562 OR Hematopoietic progenitor cells OR Monocyte-derived dendritic cells not otherwise specified OR Other Teratocarcinoma Cell Lines OR Activated helper T cells OR Brainstem OR Other Lymphoma Cell Lines OR Ovarian Cancer Cell Lines not otherwise specified OR Cell Line not otherwise specified OR NCI-H23 OR J-774A.1 OR Effector memory cytotoxic T cells OR Other Monocyte-derived dendritic cells OR A375 OR Chondrocytes OR Pancreas OR Pheochromocytoma cell lines not otherwise specified OR Salivary Gland OR Other Organ Systems OR SK-N-SH OR Naive helper T cells OR Peripheral blood lymphocytes OR SK-MEL-5 OR Large Intestine OR Corpus Callosum OR Other Bone marrow cells OR Dendritic cells not otherwise specified OR Jurkat OR Stomach OR Thalamus OR NCI-ADR-RES OR Other CNS Cell Lines OR Brain OR NCI-H522 OR Other Prostate Cancer Cell Lines OR Activated CD56bright NK cells OR HEL OR B lymphocytes not otherwise specified OR Adipose OR Other Granulocytes OR Mesenchymal stem cells OR Caco2 cells OR Skin OR Th17 cells OR Epithelial cells not otherwise specified OR Peripheral blood leukocytes not otherwise specified OR Activated Vd1 Gamma-delta T cells OR HuH7 OR Granule Cell Layer OR Lymphocytes not otherwise specified OR Swiss 3T3 cells OR U266 OR 293 cells OR Monocyte-derived macrophage OR Other Neurons OR Macrophage Cancer Cell Lines not otherwise specified OR MDA-MB-468 OR Other Nervous System OR Pancreatic Cancer Cell Lines not otherwise specified OR Other Myeloma Cell Lines OR Other Hepatoma Cell Lines OR RAW 264.7 OR Purkinje cells OR Other Osteosarcoma Cell Lines OR Cardiomyocytes OR MEF cells OR Peripheral blood monocytes OR Breast Cancer Cell Lines not otherwise specified OR Langerhans cells OR SF-295 OR HCT-116 OR HS 578T OR Bone marrow cells not otherwise specified OR HOP-92 OR NCI-H226 OR HCC-2998 OR Heart OR Other Neuroblastoma Cell Lines OR Gray Matter OR Monocytes not otherwise specified OR SK-OV-3 OR Vd1 Gamma-delta T cells OR MDA-MB-435 OR A498 OR CAKI-1 OR NIH/3T3 cells OR Pro-B lymphocytes OR Melanoma Cell Lines not otherwise specified OR J774 OR Kidney OR Megakaryocytes OR Cerebellum OR Microvascular endothelial cells OR ACHN OR WEHI-231 OR THP-1 OR Leukemia Cell Lines not otherwise specified OR CNS Cell Lines not otherwise specified OR DU-145 OR Oocytes OR Other Endothelial cells OR Other Breast Cancer Cell Lines OR Peritoneal macrophages OR Other Lymphocytes OR Esophagus OR Other NK cells OR Prostate Gland OR HT29 OR M14 OR Cortical neurons OR Other Smooth muscle cells OR Trachea OR Olfactory Bulb OR Testis OR SF-268 OR Other Colon Cancer Cell Lines OR Other Pheochromocytoma cell lines OR PBMCs OR HL-60 OR Cartilage Tissue OR Immune cell lines not otherwise specified OR HeLa OR Dermis OR Memory B cells OR Thyroid Gland OR Sciatic Nerve OR Liver OR Crypt OR Pyramidal neurons OR CD4+ T-lymphocytes OR CCRF-CEM OR Cervical cancer cell line not otherwise specified OR U937 OR Natural T-regulatory cells OR Mammary Gland OR COLO205 OR U87MG OR Calvaria OR MDA-MB-231 OR Naive B cells OR Osteoblasts OR T47-D OR

(c) 2000-2021 QIAGEN. All rights reserved.

Parietal Lobe OR Kidney cell lines not otherwise specified OR NK cells not otherwise specified OR Activated Vd2 Gamma-delta T cells OR Other Macrophage Cancer Cell Lines OR Substantia Nigra OR KM-12 OR Eosinophils OR Other T lymphocytes OR Osteosarcoma Cell Lines not otherwise specified OR INS-1 OR Hep3B OR Effector memory RA+ cytotoxic T cells OR Other Immune cell lines OR Prostate Cancer Cell Lines not otherwise specified OR LNCaP cells OR Other Macrophages OR Adrenal Gland OR Myeloid dendritic cells OR Myeloma Cell Lines not otherwise specified OR Bladder OR Other Cervical cancer cell line OR RXF-393 OR P19 OR CD56bright NK cells OR MALME-3M OR Cerebral Cortex OR UACC-257 OR H460 OR Uterus OR SNB-75 OR Dorsal Root Ganglion OR Thymus OR Other Pancreatic Cancer Cell Lines OR Epidermis OR SR OR Macrophages not otherwise specified OR Other Lung Cancer Cell Lines OR Other Monocytes OR Microglia OR Teratocarcinoma Cell Lines not otherwise specified OR Choroid Plexus OR PANC-1 OR SW-480 OR Intraepithelial T lymphocytes OR HMC-1 OR Th1 cells OR HCT-15 OR Pituitary Gland OR Cerebral Ventricles OR Other Peripheral blood leukocytes OR Small Intestine OR Vascular smooth muscle cells OR SK-MEL-28 OR UO-31 OR A2780 OR Astrocytes OR Tissues and Primary Cells not otherwise specified OR Blood platelets OR BDCA-3+ dendritic cells OR BT-474 OR IGROV1 OR Neurons not otherwise specified OR Other Immune cells OR Putamen OR BA/F3 OR Bone marrow-derived dendritic cells OR Smooth muscle cells not otherwise specified OR 786-0 OR Min6 OR OVCAR-5 OR Other Tissues and Primary Cells OR HOP-62 OR Adipocytes OR RKO OR Pre-B lymphocytes OR OVCAR-3 OR Organ Systems not otherwise specified OR NT2/D1 OR Immature monocyte-derived dendritic cells OR Keratinocytes OR Ovary OR SW-620 OR Lung OR Other Leukemia Cell Lines OR Spinal Cord OR Other Fibroblast cell lines OR Endothelial cells not otherwise specified OR Amygdala OR Plasmacytoid dendritic cells OR Ventricular Zone OR Mast cells OR Mononuclear leukocytes not otherwise specified OR 3T3-L1 cells OR Effector T cells OR Kidney Cancer Cell Lines not otherwise specified OR Other Kidney cell lines OR Spleen OR Other Ovarian Cancer Cell Lines OR Hepatocytes OR Activated CD56dim NK cells OR Nervous System not otherwise specified OR Hippocampus OR Mature monocyte-derived dendritic cells OR PC-3 OR Smooth Muscle OR Splenocytes OR MCF7 OR HepG2 OR Immune cells not otherwise specified OR Retina OR Hypothalamus OR Stromal cells OR Subventricular Zone OR Stem cells not otherwise specified OR UACC-62 OR HUVEC cells OR Embryonic stem cells OR Other B lymphocytes OR NB4 OR Other Epithelial cells OR A549-ATCC OR Memory T lymphocytes not otherwise specified OR Hepatoma Cell Lines not otherwise specified OR Thymocytes OR Fibroblast cell lines not otherwise specified OR PC-12 cells OR MDA-MB-361 OR Other Kidney Cancer Cell Lines OR Central memory helper T cells OR Lymphoma Cell Lines not otherwise specified OR Neuroblastoma Cell Lines not otherwise specified OR Effector memory helper T cells OR Sertoli cells OR NCI-H332M OR U2OS OR BDCA-1+ dendritic cells) AND (mol. types = biologic drug OR canonical pathway OR chemical - endogenous mammalian OR chemical - endogenous non-mammalian OR chemical - kinase inhibitor OR chemical - other OR chemical - protease inhibitor OR chemical drug OR chemical reagent OR chemical toxicant OR complex OR cytokine OR disease OR enzyme OR function OR fusion gene/product OR G-protein coupled receptor OR group OR growth factor OR ion channel OR kinase OR ligand-dependent nuclear receptor OR mature microRNA OR microRNA OR other OR peptidase OR phosphatase OR transcription regulator OR translation regulator OR transmembrane receptor OR transporter) AND

(c) 2000-2021 QIAGEN. All rights reserved.

(data sources = An Open Access Database of Genome-wide Association Results OR BIND OR BioGRID OR Catalogue Of Somatic Mutations In Cancer (COSMIC) OR Chemical Carcinogenesis Research Information System (CCRIS) OR Clinical Genome Resource (ClinGen) OR ClinicalTrials.gov OR ClinVar OR Cognia OR DIP OR DrugBank OR Gene Ontology (GO) OR GVK Biosciences OR Hazardous Substances Data Bank (HSDB) OR HumanCyc OR Ingenuity Expert Findings OR Ingenuity ExpertAssist Findings OR IntAct OR Interactome studies OR MIPS OR miRBase OR miRecords OR Mouse Genome Database (MGD) OR Obesity Gene Map Database OR Online Mendelian Inheritance in Man (OMIM) OR TarBase OR TargetScan Human)

Top Canonical Pathways

Name	p-value	Overlap
**Glioma Signaling**	4.67E-05	3.2 % 4/124
**Cancer Drug Resistance By Drug Efflux**	1.12E-04	5.2 % 3/58
**ERB2-ERBB3 Signaling**	1.57E-04	4.6 % 3/65
**Glioblastoma Multiforme Signaling**	1.62E-04	2.3 % 4/171
**Tumor Microenvironment Pathway**	1.93E-04	2.2 % 4/179

Top Upstream Regulators

Upstream Regulators

Name	p-value	Predicted Activation
**miR-486-5p (and other miRNAs w/seed CCUGUAC)**	4.24E-13	
**mir-486**	6.05E-13	
**FOXO1**	1.31E-05	

(c) 2000-2021 QIAGEN. All rights reserved.

**AR**	1.77E-05
**HGF**	1.88E-05

Causal Network

Name	p-value	Predicted Activation
**miR-486-5p (and other miRNAs w/seed CCUGUAC)**	4.24E-13	
**mir-486**	6.05E-13	
**MCRS1**	3.14E-08	
**CDC20**	5.97E-08	
**HUWE1**	3.63E-07	

Top Diseases and Bio Functions

Diseases and Disorders

Name	p-value range	# Molecules
**Cardiovascular Disease**	1.61E-03 - 1.06E-06	14
**Cancer**	4.82E-03 - 1.55E-06	38
**Organismal Injury and Abnormalities**	4.82E-03 - 1.55E-06	38
**Renal and Urological Disease**	4.82E-03 - 7.57E-06	16
**Hematological Disease**	4.82E-03 - 1.68E-05	19

(c) 2000-2021 QIAGEN. All rights reserved.

INGENUITY PATHWAY ANALYSIS

Molecular and Cellular Functions

Name	p-value range	# Molecules
Cellular Development	4.82E-03 - 7.57E-06	17
Cellular Function and Maintenance	4.82E-03 - 7.57E-06	14
Cellular Growth and Proliferation	4.82E-03 - 7.57E-06	15
Cell Morphology	4.82E-03 - 1.51E-05	18
Cell Cycle	4.82E-03 - 2.52E-05	10

Physiological System Development and Function

Name	p-value range	# Molecules
Cardiovascular System Development and Function	4.53E-03 - 1.06E-06	12
Embryonic Development	4.82E-03 - 1.06E-06	13
Organ Development	3.95E-03 - 1.06E-06	12
Organismal Development	4.53E-03 - 1.06E-06	18
Tissue Development	4.82E-03 - 1.06E-06	16

Top Tox Functions

Assays: Clinical Chemistry and Hematology

Name	p-value range	# Molecules
Increased Levels of Albumin	1.12E-02 - 1.12E-02	1

(c) 2000-2021 QIAGEN. All rights reserved.

**Increased Levels of Red Blood Cells**	1.61E-01 - 1.61E-01	1

Cardiotoxicity

Name	p-value range	# Molecules
**Cardiac Enlargement**	2.54E-01 - 2.01E-04	7
**Pulmonary Hypertension**	1.61E-03 - 1.37E-03	2
**Cardiac Damage**	1.61E-03 - 1.61E-03	1
**Cardiac Dilation**	3.94E-02 - 1.61E-03	3
**Cardiac Necrosis/Cell Death**	6.40E-02 - 1.61E-03	3

Hepatotoxicity

Name	p-value range	# Molecules
**Liver Proliferation**	9.08E-02 - 3.95E-03	3
**Liver Hyperplasia/Hyperproliferation**	4.35E-01 - 4.82E-03	19
**Liver Regeneration**	8.48E-03 - 8.48E-03	2
**Liver Steatosis**	1.44E-01 - 8.84E-03	4
**Liver Damage**	9.62E-03 - 9.62E-03	1

Nephrotoxicity

Name	p-value range	# Molecules
**Glomerular Injury**	5.64E-02 - 1.61E-03	2

(c) 2000-2021 QIAGEN. All rights reserved.

INGENUITY PATHWAY ANALYSIS

**Renal Hypoplasia**	6.43E-03 - 6.43E-03	1
**Renal Inflammation**	1.60E-02 - 1.60E-02	1
**Renal Nephritis**	1.60E-02 - 1.60E-02	1
**Renal Enlargement**	2.55E-02 - 2.55E-02	1

Top Regulator Effect Networks

Top Networks

ID	Associated Network Functions	Score
**1**	Cancer, Organismal Injury and Abnormalities, Renal and Urological Disease	39
**2**	Cell Signaling, Molecular Transport, Vitamin and Mineral Metabolism	19
**3**	Cellular Function and Maintenance, Hypersensitivity Response, Inflammatory Response	14
**4**	Organ Development, Reproductive System Development and Function, Amino Acid Metabolism	12

(c) 2000-2021 QIAGEN. All rights reserved.

Top Tox Lists

Name	p-value	Overlap
**Cardiac Hypertrophy**	5.34E-04	1.2 % 5/419
**Cell Cycle: G1/S Checkpoint Regulation**	5.38E-03	2.9 % 2/68
**Liver Proliferation**	8.84E-03	1.1 % 3/265
**Cardiac Necrosis/Cell Death**	1.42E-02	0.9 % 3/316
**Nongenotoxic Hepatocarcinogenicity Biomarker Panel**	3.49E-02	4.5 % 1/22

Top My Lists

Top My Pathways

Top Analysis-Ready Molecules

(c) 2000-2021 QIAGEN. All rights reserved.

INGENUITY
PATHWAY ANALYSIS
