# Supplementary material for: Association of microRNAs With Embryo Development and Fertilization in Women Undergoing Subfertility Treatments: A Pilot Study
Source: Front Reprod Health. 2021 Sep 23;3:719326. doi: 10.3389/frph.2021.719326 (PMC9580729; doi:10.3389/frph.2021.719326)
Supplement: Supplementary file 4 [file Table_1.DOCX]

**Supplementary Table 1.** MicroRNA panel (n=176) used in the study in the 48 women undergoing in vitro fertilisation.

|  |
| --- |
| hsa-miR-1260a |
| hsa-miR-18b-5p |
| hsa-miR-424-5p |
| hsa-let-7b-3p |
| hsa-miR-2110 |
| hsa-let-7c-5p |
| hsa-miR-454-3p |
| hsa-miR-130a-3p |
| hsa-miR-150-5p |
| hsa-miR-133b |
| hsa-miR-362-3p |
| hsa-miR-205-5p |
| hsa-miR-125a-5p |
| hsa-miR-22-3p |
| hsa-miR-29c-3p |
| hsa-miR-375 |
| hsa-let-7b-5p |
| hsa-miR-342-3p |
| hsa-miR-136-3p |
| hsa-miR-30d-5p |
| hsa-miR-23a-3p |
| hsa-miR-18a-5p |
| hsa-miR-339-3p |
| hsa-miR-185-5p |
| hsa-miR-200c-3p |
| hsa-miR-99b-5p |
| hsa-miR-20b-5p |
| hsa-miR-222-3p |
| hsa-miR-874-3p |
| hsa-miR-210-3p |
| hsa-miR-484 |
| hsa-miR-26b-5p |
| hsa-miR-425-3p |
| hsa-miR-132-3p |
| hsa-miR-502-3p |
| hsa-miR-133a-3p |
| hsa-miR-328-3p |
| hsa-miR-423-3p |
| hsa-miR-324-5p |
| hsa-miR-361-5p |
| hsa-let-7d-5p |
| hsa-miR-766-3p |
| hsa-miR-33a-5p |
| hsa-miR-29a-3p |
| hsa-miR-140-5p |
| hsa-miR-192-5p |
| mmu-miR-378a-3p |
| hsa-miR-145-5p |
| hsa-miR-146b-5p |
| hsa-miR-106b-3p |
| hsa-miR-324-3p |
| hsa-let-7a-5p |
| hsa-miR-374b-5p |
| hsa-miR-374a-5p |
| hsa-miR-26a-5p |
| hsa-miR-181a-5p |
| hsa-miR-223-3p |
| hsa-miR-382-5p |
| hsa-miR-590-5p |
| hsa-miR-15b-3p |
| hsa-miR-148a-3p |
| hsa-miR-148b-3p |
| hsa-let-7i-5p |
| hsa-miR-34a-5p |
| hsa-miR-7-5p |
| hsa-miR-193a-5p |
| hsa-miR-10b-5p |
| hsa-miR-338-3p |
| hsa-miR-16-5p |
| hsa-let-7g-5p |
| hsa-miR-19a-3p |
| hsa-miR-99a-5p |
| hsa-let-7e-5p |
| hsa-miR-7-1-3p |
| hsa-miR-151a-5p |
| hsa-miR-505-3p |
| hsa-miR-130b-3p |
| hsa-miR-92b-3p |
| hsa-miR-532-5p |
| hsa-miR-106a-5p |
| hsa-miR-122-5p |
| hsa-miR-21-5p |
| hsa-miR-140-3p |
| hsa-miR-23b-3p |
| hsa-miR-423-5p |
| hsa-miR-501-3p |
| hsa-miR-543 |
| hsa-miR-125b-5p |
| hsa-miR-142-5p |
| hsa-miR-30e-5p |
| hsa-let-7f-5p |
| hsa-miR-660-5p |
| hsa-miR-197-3p |
| hsa-miR-335-5p |
| hsa-miR-151a-3p |
| hsa-miR-24-3p |
| hsa-miR-363-3p |
| hsa-miR-19b-3p |
| hsa-miR-339-5p |
| hsa-miR-29b-3p |
| hsa-miR-128-3p |
| hsa-miR-495-3p |
| hsa-miR-22-5p |
| hsa-miR-320c |
| hsa-miR-127-3p |
| hsa-miR-194-5p |
| hsa-miR-320a |
| hsa-miR-101-3p |
| hsa-miR-584-5p |
| hsa-miR-200a-3p |
| hsa-miR-885-5p |
| hsa-miR-409-3p |
| hsa-miR-100-5p |
| hsa-miR-15b-5p |
| hsa-miR-376a-3p |
| hsa-miR-146a-5p |
| hsa-miR-421 |
| hsa-let-7d-3p |
| hsa-miR-92a-3p |
| hsa-miR-126-5p |
| hsa-miR-30a-5p |
| hsa-miR-326 |
| hsa-miR-215-5p |
| hsa-miR-30e-3p |
| hsa-miR-28-5p |
| hsa-miR-106b-5p |
| hsa-miR-142-3p |
| hsa-miR-320b |
| hsa-miR-486-5p |
| hsa-miR-191-5p |
| hsa-miR-877-5p |
| hsa-miR-195-5p |
| hsa-miR-136-5p |
| hsa-miR-223-5p |
| hsa-miR-451a |
| hsa-miR-574-3p |
| hsa-miR-141-3p |
| hsa-miR-126-3p |
| hsa-miR-25-3p |
| hsa-miR-199a-3p |
| hsa-miR-155-5p |
| hsa-miR-652-3p |
| hsa-miR-154-5p |
| hsa-miR-28-3p |
| hsa-miR-629-5p |
| hsa-miR-425-5p |
| hsa-miR-103a-3p |
| hsa-miR-144-3p |
| hsa-miR-15a-5p |
| hsa-miR-17-5p |
| hsa-miR-32-5p |
| hsa-miR-221-3p |
| hsa-miR-30c-5p |
| hsa-miR-376c-3p |
| hsa-miR-186-5p |
| hsa-miR-27a-3p |
| hsa-miR-93-3p |
| hsa-miR-365a-3p |
| hsa-miR-93-5p |
| hsa-miR-1 |
| hsa-miR-107 |
| hsa-miR-144-5p |
| hsa-miR-152-3p |
| hsa-miR-139-5p |
| hsa-miR-27b-3p |
| hsa-miR-335-3p |
| hsa-miR-30b-5p |
| hsa-miR-16-2-3p |
| hsa-miR-320d |
| hsa-miR-301a-3p |
| hsa-miR-143-3p |
| hsa-miR-485-3p |
| hsa-miR-532-3p |
| hsa-miR-199a-5p |
| hsa-miR-331-3p |
| hsa-miR-497-5p |
| hsa-miR-20a-5p |
